# Gitelman syndrome in a South African family presenting with hypokalaemia and unusual food cravings

**DOI:** 10.1186/s12882-017-0455-3

**Published:** 2017-01-26

**Authors:** Pieter Du Toit van der Merwe, Megan A. Rensburg, William L. Haylett, Soraya Bardien, M. Razeen Davids

**Affiliations:** 1 Division of Nephrology, Department of Medicine, Stellenbosch University and Tygerberg Hospital, Cape Town, South Africa; 2 Division of Chemical Pathology, Stellenbosch University and National Health Laboratory Service, Cape Town, South Africa; 3 Division of Molecular Biology and Human Genetics, Stellenbosch University, Cape Town, South Africa

**Keywords:** South Africa, Gitelman syndrome, Hypokalaemia, Tubular disorders, Salt craving, Mutation, Pseudo-dominant inheritance

## Abstract

**Background:**

Gitelman syndrome (GS) is an autosomal recessive renal tubular disorder characterised by renal salt wasting with hypokalaemia, metabolic alkalosis, hypomagnesaemia and hypocalciuria. It is caused by mutations in *SLC12A3* encoding the sodium-chloride cotransporter on the apical membrane of the distal convoluted tubule. We report a South African family with five affected individuals presenting with hypokalaemia and unusual food cravings.

**Methods:**

The affected individuals and two unaffected first degree relatives were enrolled into the study. Phenotypes were evaluated through history, physical examination and biochemical analysis of blood and urine. Mutation screening was performed by sequencing of *SLC12A3*, and determining the allele frequencies of the sequence variants found in this family in 117 ethnically matched controls.

**Results:**

The index patient, her sister, father and two aunts had a history of severe salt cravings, fatigue and tetanic episodes, leading to consumption of large quantities of salt and vinegar. All affected individuals demonstrated hypokalaemia with renal potassium wasting. Genetic analysis revealed that the pseudo-dominant pattern of inheritance was due to compound heterozygosity with two novel mutations: a S546G substitution in exon 13, and insertion of AGCCCC at c.1930 in exon 16. These variants were present in the five affected individuals, but only one variant each in the unaffected family members. Neither variant was found in any of the controls.

**Conclusions:**

The diagnosis of GS was established in five members of a South African family through clinical assessment, biochemical analysis and mutation screening of the *SLC12A3* gene, which identified two novel putative pathogenic mutations.

## Background

Gitelman syndrome (GS) is an autosomal recessive renal tubular disorder which is characterised by renal salt wasting with hypokalaemia, metabolic alkalosis, hypomagnesaemia and hypocalciuria [1]. It is caused by mutations in the solute carrier family 12, member 3 gene (*SLC12A3*) encoding the sodium-chloride cotransporter (NCC) of the distal convoluted tubule (DCT) [2–4]. Although a proportion of patients with GS have homozygous mutations, the majority are compound heterozygotes, with a different mutation on each allele [5]. It is therefore not uncommon to encounter affected cases in successive generations of the same family [6, 7], giving rise to a pseudo-dominant pattern of inheritance [7, 8].

GS usually presents during adolescence or adulthood, although it may be completely asymptomatic for life. Common symptoms include cramps or tetany, paraesthesias, lethargy, muscle weakness [2] and, rarely, periodic paralysis [9]. Polydipsia and nocturia commonly occur, as well as cravings for salty substances [10–13]. Uncommonly, patients may have growth retardation, delayed puberty [14], cardiac dysrhythmias or sudden cardiac death [15–17]. Diabetes mellitus and chronic kidney disease have been reported with higher frequencies in GS than in the general population [8]. A large amount of phenotype variability occurs, with poor correlation between specific mutations and clinical manifestations [18, 19]. Moreover, features such as hypokalaemia and hypomagnesaemia may change throughout the course of one individual’s life. Differences in dietary habits or gender [20] may contribute to phenotype variability, but in most cases the reasons are unknown.

The diagnosis of GS is based on the typical symptoms and biochemical abnormalities. It is distinguished from Bartter syndrome (BS) by a low rate of calcium excretion (urine calcium/creatinine ratio ≤0.1 mmol/mmol for GS versus >0.1 mmol/mmol for BS) [10, 21, 22]. Genetic analysis is a valuable tool in assisting with the diagnosis of GS, but is available only at specialised centres [23].

In this paper we describe the clinical, biochemical and genetic features of a South African family with GS. The family is interesting in that several individuals across two generations were affected, the affected individuals reported unusual food cravings, and two novel putative pathogenic mutations were identified.

## Methods

### Clinical assessment

The index patient was referred to our nephrology clinic at Tygerberg Hospital (Cape Town, South Africa) with the problem of persistent hypokalaemia. She gave a history of several family members with similar symptoms. These individuals, along with the index patient’s unaffected mother and brother, agreed to be studied to assist with the diagnosis of a suspected renal tubular disorder. The index patient, her sister and father were examined at our clinic at the start of the study. They were then followed up along with the other study participants (except for the father, who had passed away in the interim) at their local general practitioners’ offices. Special emphasis was placed on obtaining a detailed history of drug use, previous and current symptoms associated with GS, as well as the presence and nature of food cravings.

**Fig. 1:**
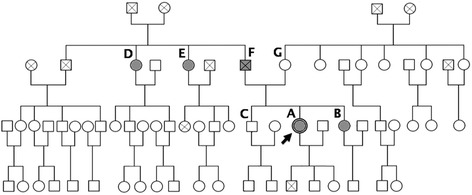
Pedigree of the extended South African family with Gitelman syndrome. Affected participants are represented by shaded symbols; the proband is indicated by an arrow; deceased individuals are shown with a cross inside the symbol

The index patient (study participant A, proband, see Fig. 1) was a white Afrikaner female from the Northern Cape province in South Africa. She was 31 years old at the commencement of the study. Her lowest recorded serum potassium level had been 2.4 mmol/L 8 years earlier. She had experienced symptoms of marked salt craving, weakness, fatigue and tetany. This presentation, along with a positive family history, led us to consider the diagnosis of GS.

She gave the history of a predilection for salt-containing and sour foods since childhood. She had developed the habit of drinking white vinegar with extra salt added. Initially she consumed half a glass of vinegar with a heaped teaspoon of salt per day, but this escalated to the point where she was drinking up to two litres of this mixture per week. More peculiarly, she would prepare ice cubes of the same mixture for later consumption. This practice was also followed by her younger sister (participant B). It emerged that her father (F) and his older sisters (D and E) had done the same when they were young, also drinking white vinegar mixed with salt. The index patient reported that she had learnt the habit from her father and aunts. They all had a fondness for sour foods, including lemons and sour figs, often with extra salt added. During their childhood, sour-tasting berries and plants were sometimes gathered and consumed. However, at the time of our study, most of the participants had outgrown these cravings. One affected individual had grown to dislike salt and sour foods and avoided them altogether.

Only the index patient (participant A) had previously had significant polydipsia, consuming up to seven litres of water a day. All five affected participants had experienced episodes of paraesthesiae, especially of the lips and hands, often culminating in overt tetany with carpopedal spasm. This was often provoked by anxiety accompanied by mild hyperventilation. Other notable symptoms reported were nausea and vomiting, fatigue, cramps, palpitations, atypical chest pain, muscular weakness and syncopal attacks (Table 1).

**Table 1:** Clinical data from members of the South African family with Gitelman syndrome

Participants	A - affected	B - affected	D - affected	E - affected	F - affected	C - unaffected	G - unaffected
Age, sex	31y, female	25y, female	75y, female	69y, female	65y, male	34y, male	61y, female
Onset of symptoms	Early adolescence	Early adolescence	Early adolescence	Early adolescence	Early adolescence	Not applicable	Not applicable
Food cravings	Salt and white vinegar, lemons, sour figs	Salt and white vinegar, lemons, sour figs	White vinegar during youth, lemons, sour-tasting field plants	Salt and white vinegar during youth	Salt and white vinegar, lemons		
Fatigue	Since adolescence	Since adolescence	If supplements omitted	If supplements omitted		Since thirties	
Tetany	Episodes of carpopedal spasm	Episodes of carpopedal spasm	Carpopedal spasm as a child	Carpopedal spasm as a child	Occasional episodes of carpopedal spasm (mild)		
Palpitations	1-2 times per week	Episodes previously		When hypokalaemic	Since myocardial infarction		Recent onset (pre-stent)
Chest pain		Accompanies palpitations		L-sided muscular pain	During youth; myocardial infarction previously		Recent onset (pre-stent)
Dizziness	Frequently during adolescence	Frequently since adolescence		Vertigo and tinnitus previously			
Syncope				During youth only		One episode during his youth	One episode during her youth
Muscle weakness or tremor	Occasional L arm weakness	Episodes of R arm weakness		Resting tremor R arm			
Cramps		Occasional			Occasional leg cramps	Occasional	
Polydipsia	Drank ± 7 L/day, now 2 L		±1 L daily, more with sport				Yes (on diuretic)
Vomiting		During pregnancy	During pregnancy	During pregnancy			
Comorbidities			Osteoarthritis		IHD	Hypertension, depression	Hypertension, IHD
Smoking	15 pack years	7 pack years	Quit 20 years ago	50 pack years	40 pack years	30 pack years	40 pack years
Height, weight	1.67 m, 96 kg	1.69 m, 83 kg	1.54 m, 72 kg	1.52 m, 63 kg	1.78 m, 70 kg	1.86 m, 110 kg	1.63 m, 94 kg
BMI kg/m^2^	34	29	30	27	22	32	35
Blood pressure	110/70 mmHg	110/60 mmHg	170/80 mmHg	140/85 mmHg	125/80 mmHg	130/80 mmHg	150/90 mmHg
Medication for GS	KCl ampoules as required	KCl ampoules as required	Slow-K® 6/day	Slow-K® 8/day, “tissue salts”	Spironolactone,^a^ losartan,^a^ Slow-K®		
Other medication	Previously on spironolactone, Slow K® and KCl ampoules	Previously on etilefrine and KCl ampoules	Analgesics (NSAIDs, tramadol, paracetamol)	Propranolol	Carvedilol, simvastatin, aspirin	Citalopram, not currently taking antihypertensives	Carvedilol, aspirin, furosemide

^a^Treatment for both heart failure and GS

*IHD* Ischaemic heart disease

All participants but one were heavy smokers and two had a history of ischaemic heart disease. Both unaffected participants had previously been diagnosed with hypertension and one affected participant had high blood pressure recorded for the first time during this study. The index patient’s paternal grandfather had died suddenly at age 36, after collapsing at work without any prior history of illness. He was presumed to have died from a myocardial infarction, but the exact cause of death was never established.

All affected female participants in our study had had one or more successful pregnancies, albeit with generally severe gestational nausea and vomiting. The only poor outcome reported was that of the index patient’s first pregnancy which ended in foetal death due to abruptio placentae at 35 weeks’ gestation. One participant experienced worsening of her food cravings during pregnancy, whereas the others reported improvement in their symptoms.

### Biochemical analysis of blood and urine

Analysis of blood and urine samples from the first three participants was done at the National Health Laboratory Service at Tygerberg Hospital. The remaining participants’ samples were analysed at PathCare laboratories. These laboratories also provided the results of previous tests done on the study participants.

### Genetic analysis

Genomic DNA was isolated from whole blood samples at the Faculty of Medicine and Health Sciences of Stellenbosch University according to established methods. Genetic screening of all 26 exons of the *SLC12A3* gene was performed in accordance with protocols described by Simon et al. [3].

Following the identification of two sequence variants in exons 13 and 16 of *SLC12A3*, the respective allele frequencies were determined in 117 ethnically matched controls by means of high-resolution melt (HRM) analysis on a RotorGene 6000 analyser (Corbett Life Science). The anonymised controls were recruited from the Western Province Blood Transfusion Service clinics in Cape Town. Samples with altered heat denaturation profiles following HRM analysis were Sanger sequenced in order to characterise the sequence variants.

## Results

### Biochemical analysis of blood and urine

The results of various biochemical analyses are provided in Table 2. Amongst the five participants suspected to have GS, four were found to be hypokalaemic. The fifth had had hypokalaemia documented previously, and was taking potassium supplementation at the time of testing for the study. The four hypokalaemic participants all had inappropriately high renal potassium excretion, with urinary K^+^:Cr ratios >1.5 mmol/mmol. Four of the five affected participants had hypocalciuria, with urinary calcium:creatinine ratios <0.1 mmol/mmol, as would be expected with GS. All participants had normal serum magnesium levels.

**Table 2:** Biochemical analysis of blood and random urine samples

Participants		A	B	D	E	F	C	G
Blood	Ref ranges/units	Affected	Affected	Affected	Affected	Affected	Unaffected	Unaffected
Sodium	136–146 mmol/L	141	143	142	140	146	137	133
Potassium (lowest)	3.5–5.1 mmol/L	2.8	2.6	2.8	2.8	2.9	3.6	4.0
Potassium (latest)	3.5–5.1 mmol/L	3.2	3.0	3.0	3.2	4.1	4.1	4.4
Chloride	101–109 mmol/L	100	100	103	101	103	102	102
Urea	2.8–7.2 mmol/L	5.8	7.7	11.7	9.9	13.5	6.4	6.4
Creatinine	59–104 μmol/L	94	82	86	92	111	88	48
eGFR	mL/min/1.73 m^2^	66^a^	82^a^	57	55	62^a^	98	102
Corrected calcium	2.20–2.65 mmol/L	2.33	2.19	2.40	2.32	2.30	2.26	2.23
Albumin	35–52 g/L	41	42	40	44	43	45	43
Magnesium	0.73–1.06 mmol/L	0.85	0.96	0.83	0.77	0.85	1.00	0.97
Phosphate	0.81–1.45 mmol/L	1.08	0.78	1.00	1.00	0.96	1.32	1.43
Alkaline phosphatase	30–120 IU/L	36	43	70	71	47	52	111
Cortisol	am 184–618; pm <276 nmol/L	291	385	203	204	318	145	125
pH	7.36–7.44	7.37	7.38	7.41	7.35	7.35	7.42	7.39
Bicarbonate	23–27 mmol/L	32.3	30.3	30.0	30.0	31.6	27.0	26.0
Renin	2.7–27.7 ng/L (erect)	46.4	40.3	9.1	41.4	68.3	10.6	Not done
Aldosterone	49–1066 pmol/L	152	146	336	357	231	273	Not done
Aldo:renin ratio	<118 pmol/ng	3.3	3.6	36.9	8.6	3.4	25.8	Not done
Urine								
FE sodium	%	1.8	1.6	0.1	0.8	0.8	0.3	0.1
FE potassium	%	17.5	25.5	18.3	53.0	23.3	5.3	3.1
FE chloride	%	1.6	2.2	0.5	2.4	1.3	0.5	0.1
FE magnesium	%	1.4	2.5	0.7	4.5	3.5	0.9	0.4
K:creat ratio	<1.5 mmol/mmol in hypokalaemia	6.0	9.3	6.4	18.4	8.6	2.5	6.4
Ca:creat ratio	mmol/mmol	0.04	0.03	0.07	0.18	0.07	0.23	0.22
Mg:creat ratio	mmol/mmol	0.13	0.03	0.07	0.38	0.27	0.10	0.08
Osmolality	mOsm/kg	813	888	638	539	1049	968	725
pH	6.0	8.0	7.0	7.5	6.5	5.0	5.0	5.0
Protein:creat ratio	<0.2 mg/mg	0.15	0.12	0.12	0.18	0.08	0.09	0.08

*eGFR* estimated glomerular filtration rate as calculated by the CKD-EPI and ^a^MDRD formulae; *FE* fractional excretion in %

The affected participants had elevated serum bicarbonate levels, with normal blood pH values, and in four of them the urine pH was >6.0. All affected participants had varying degrees of renal function impairment, two with an estimated glomerular filtration rate (eGFR) below 60 ml/min/1.73 m^2^. All affected participants except one (whose blood pressure was elevated at the time of testing) had elevated renin activity, but normal serum aldosterone levels and normal aldosterone:renin ratios.

### Genetic analysis

**Fig. 2:**
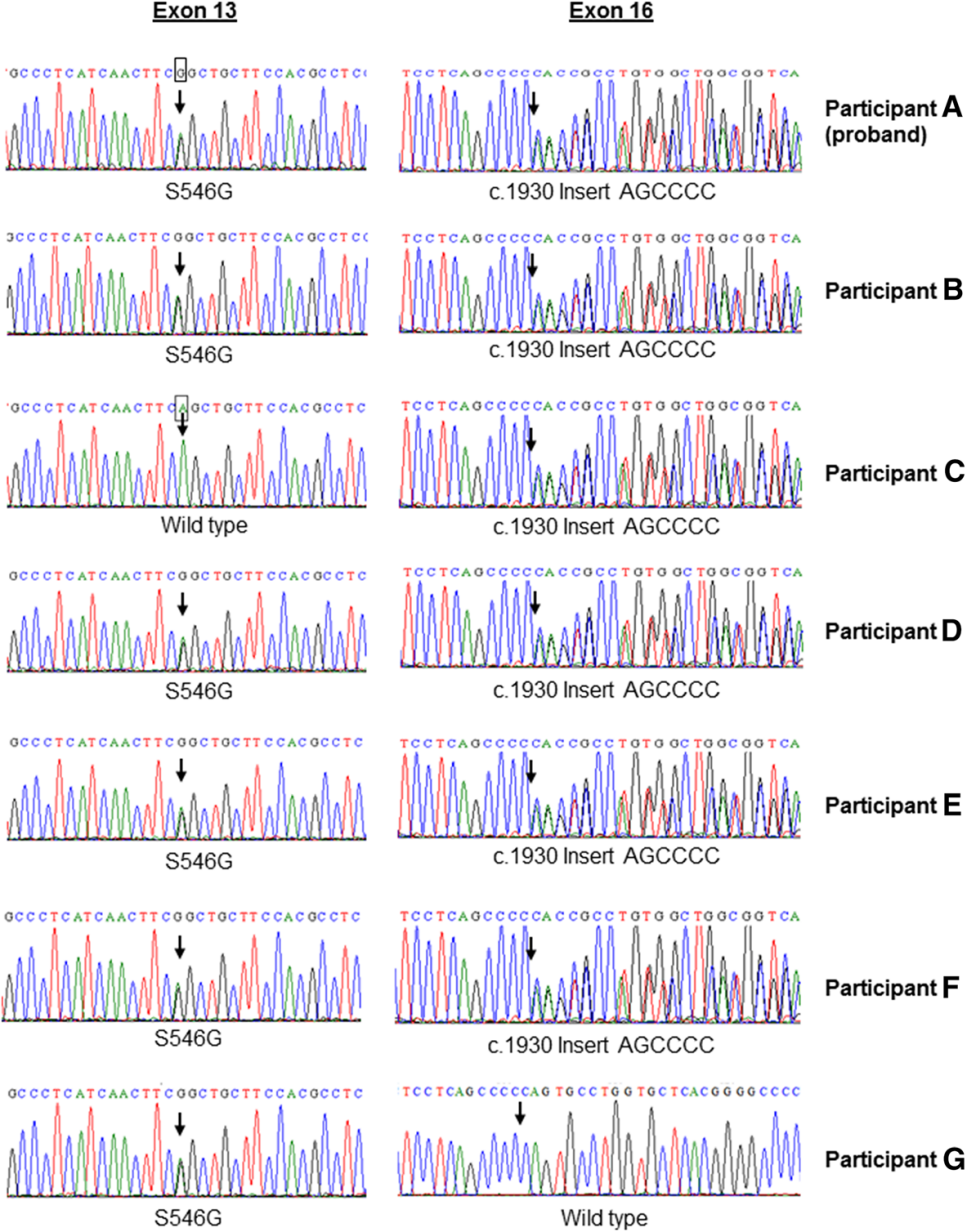
Sequence chromatograms of the*SLC12A3* gene of the family with Gitelman syndrome, indicating the presence of the two mutations S546G and c.1930insAGCCCC. The proband is indicated as participant A. Participants B, D, E and F are affected family members, while participants C and G are clinically unaffected

Genetic analysis of the affected participants identified several sequence variants in the *SLC12A3* gene (Fig. 2). All five affected individuals were found to harbour compound heterozygous putative mutations consisting of an AGC>GGC missense variant in exon 13 (exon 13+69A>G; p.S546G; ss2137143999) and an insertion of six bases (AGCCCC) in exon 16 (c.1930insAGCCCC; ss2137144000). These two heterozygous sequence variants, when compounded, would be expected to alter the protein structure and could thus be implicated in loss of function of the NCC. Unaffected participant G was found to be heterozygous for the S546G variant without the presence of an additional variant. Similarly, unaffected participant C possessed only the heterozygous c.1930insAGCCCC variant.

Neither of these variants are found in the ExAC database (http://exac.broadinstitute.org/), and, to our knowledge, have not been described previously. The frequencies of the p.S546G and c.1930insAGCCCC variants were determined in unrelated, ethnically matched controls. Neither variant was observed in any of the 117 controls (234 chromosomes) screened.

Sequence analysis also identified additional variants in the proband, including a previously described single nucleotide polymorphism (SNP) in exon 6 in the heterozygous state (rs1529927; p.A264G), and three intronic SNPs, namely homozygous IVS14-8T>C, heterozygous IVS24-13T>C and heterozygous IVS25+13C>T.

## Discussion

Our clinical suspicion of GS in five members of a South African family has been confirmed. We have characterised the phenotype of these individuals through careful clinical evaluation and biochemical analysis of blood and urine. We have also characterised their genotype, identifying compound heterozygosity due to two novel mutations in the *SLC12A3* gene in each of the affected individuals. This was responsible for the pseudo-dominant pattern of inheritance observed in this family.

The striking cravings for salt and vinegar are likely a response to the volume depletion induced by renal sodium wasting. The intake of such large amounts of vinegar is unusual, though, and seldom described in patients with GS. We have come across one other paper reporting patients with GS drinking the vinegar from pickle jars [24].

Although GS is generally considered to be a benign condition, there have been isolated reports suggesting a link with sudden cardiac death [15–17]. The proposed mechanism is thought to be diminished cardiovascular responsiveness to stress such as strenuous exercise [16]. The family history revealed sudden death at a young age of the index patient’s paternal grandfather. There is, however, no indication as to whether he had had symptoms of GS, and an exact cause of death was never established. The index patient’s father (participant F) also suffered suspected sudden cardiac death after collapse without warning symptoms. He, however, was 65 years old and had a clear history of severe coronary artery disease.

The index patient’s first pregnancy ended in foetal death due to abruptio placentae at 35 weeks’ gestation. Her smoking history may have been a contributory factor. Pregnancy outcomes are generally favourable in patients with GS if adequate electrolyte supplementation is taken [25].

Clinical examination revealed normotension in the affected participants except for participant D, whose blood pressure was elevated at the time of examination. Although GS is characterised by normotension during youth, it does not preclude the development of hypertension during later life. It has even been proposed that individuals with GS may be predisposed to the development of hypertension due to chronic vasoconstriction brought about by the compensatory hyperreninaemic state, eventually causing hypertension to supervene once hypovolaemia is overcome [24]. However, there is lack of consensus over the degree to which the NCC is involved in determining blood pressure levels, with evidence for [26] and against this role [27].

Hypokalaemia had been well documented in all the affected participants, along with evidence of inappropriate urinary potassium losses. All affected participants had metabolic alkalosis as well as hypocalciuria. They were all normomagnesaemic. Although hypomagnesaemia is common in GS, normal magnesium levels are seen in 20–40% of cases [28, 29] and may indicate a milder manifestation of the disease [30, 31]. It is possible that hypomagnesaemia may have been present in our participants when they were experiencing more pronounced symptoms, notably the episodes of tetany. Hypomagnesaemia is strongly implicated in the pathogenesis of tetany in GS [32], although the hypokalaemia and alkalosis may also be responsible.

All affected participants had some degree of impaired renal function, with two having eGFR values below 60 ml/min/1.73 m^2^. These individuals were 74 and 69 years old respectively. The reason for their impaired renal function was not clear, but one participant had a history of recurrent urinary tract infections in the years following childbirth, necessitating pelvic floor surgery. She also had a history of NSAID use for chronic lower back pain caused by a previous sports injury. No participant reported any history of kidney stones, a common complication often associated with renal impairment. In contrast to BS, GS has not been directly associated with renal impairment [33] although isolated cases have been reported in the literature [34].

Genetic studies in the proband confirmed the diagnosis of GS by demonstrating two novel putative loss-of-function mutations in the *SLC12A3* gene. Both these mutations were also present in the other affected participants. The two unaffected participants each had one of the mutations. This suggests that compound heterozygosity is responsible for the phenotype observed in our affected cohort, i.e. the two mutations occur on different alleles. Additional support for the pathogenicity of these variants is provided by the prediction website MutationTaster (http://www.mutationtaster.org), which predicts both to be “disease causing” with probability values of 0.99 and 1 for S546G and c.1930insAGCCCC, respectively [35]. Also, the PolyPhen-2 tool, which predicts the possible impact of an amino acid substitution (http://genetics.bwh.harvard.edu/pph2/index.shtml), predicts S546G to be “possibly damaging” with a score of 0.603. Another intriguing observation was that S546G was present in both the affected father and the unaffected mother of the proband. This raises the possibility of consanguinity, although it could not be established within at least the preceding three generations.

## Conclusions

We have made a clinical diagnosis of GS in a South African family with chronic hypokalaemia and typical symptoms. We have confirmed the diagnosis by genetic analysis and demonstrated two novel mutations in the *SLC12A3* gene in five affected individuals, and in two family members not clinically affected who harboured one mutation each. Several local studies have researched mutations implicated in other tubulopathies, notably Liddle syndrome [36–39]. However, this study, to our knowledge, is the first to characterise a family with GS in Southern Africa.

## Abbreviations

A: Adenine
BS: Bartter syndrome
C: Cytosine
c.1930insAGCCCC: Insertion of AGCCCC at coding DNA position 1930
CKD-EPI: Chronic Kidney Disease Epidemiology Collaboration
dbSNP: The Single Nucleotide Polymorphism database of the NCBI
DCT: Distal convoluted tubule
DNA: Deoxyribonucleic acid
eGFR: Estimated glomerular filtration rate
FE: Fractional excretion
G: Guanine
GS: Gitelman syndrome
HRM: High-resolution melt
IHD: Ischaemic heart disease
IVS14-8 T > C: Thymine to cytosine substitution at intervening sequence 14–8
IVS24-13 T > C: Thymine to cytosine substitution at intervening sequence 24–13
IVS25 + 13C > T: Cytosine to thymine substitution at intervening sequence 25 + 13
K^+^:Cr: Potassium to creatinine ratio
MDRD: Modification of Diet in Renal Disease
mmHg: Millimetres of mercury
mmol/mmol: Millimoles per millimole
mOsm: Milliosmoles
NCBI: National Center for Biotechnology Information
NCC: Sodium-chloride cotransporter
NSAIDs: Non-steroidal anti-inflammatory drugs
p.A264G: Alanine to glycine substitution at protein sequence position 264
p.S546G (S546G): Serine to glycine substitution at protein sequence position 546
rs: Reference SNP number
*SLC12A3*: Solute carrier family 12, member 3 gene
SNP: Single nucleotide polymorphism
ss: Submitted SNP number
T: Thymine.
